# Optical Properties of Complex Plasmonic Materials Studied with Extended Effective Medium Theories Combined with Rigorous Coupled Wave Analysis

**DOI:** 10.3390/ma11030351

**Published:** 2018-02-27

**Authors:** Elie Nadal, Noémi Barros, Hervé Glénat, Hamid Kachakachi

**Affiliations:** 1 University of Perpignan Via Domitia (UPVD), 52 Avenue Paul Alduy, 66100 Perpignan, France; noemi.barros@univ-perp.fr (N.B.); hamid.kachkachi@promes.cnrs.fr (H.K.); 2 Processes, Materials, and Solar Energy Laboratory, CNRS (PROMES-CNRS, UPR 8521), Rambla de la Thermodynamique, 66100 Perpignan, France; herve.glenat@univ-perp.fr

**Keywords:** gold nanoparticles, plasmon, nanocomposites, laser interferometry, Maxwell–Garnett theory, gratings, diffraction, RCWA

## Abstract

In this study we fabricate gold nanocomposites and model their optical properties. The nanocomposites are either homogeneous films or gratings containing gold nanoparticles embedded in a polymer matrix. The samples are fabricated using a recently developed technique making use of laser interferometry. The gratings present original plasmon-enhanced diffraction properties. In this work, we develop a new approach to model the optical properties of our composites. We combine the extended Maxwell–Garnett model of effective media with the Rigorous Coupled Wave Analysis (RCWA) method and compute both the absorption spectra and the diffraction efficiency spectra of the gratings. We show that such a semi-analytical approach allows us to reproduce the original plasmonic features of the composites and can provide us with details about their inner structure. Such an approach, considering reasonably high particle concentrations, could be a simple and efficient tool to study complex micro-structured system based on plasmonic components, such as metamaterials.

## 1. Introduction

Complex plasmonic systems, and in particular plasmonic nanocomposites, have emerged as promising materials for numerous applications in photonics [1,2,3], photovoltaics [4,5,6,7], and in environmental issues such as water remediation [8,9,10] or hydrogen production through water splitting [11,12,13]. Indeed, due to their plasmonic features, these systems exhibit novel optical as well as electrical properties. The control of these features, with the purpose to design functional materials or devices with tailored functionalities, often requires an organization of the metallic nanostructures into specific arrangements [14]. For instance, in photonics, the design of metamaterials, defined as exotic materials producing non-natural optical properties, mostly relies on the ability to create complex and regular 3D composite superlattices [1,3,15]. The insertion of plasmonic nanostructures in organic solar cells has also been demonstrated as an efficient way to improve the conversion efficiency of these devices. Once again, the different scenarios used to enhance the optical properties of the system strongly rely on the ability to precisely insert the metallic particles, acting as absorbers or diffusers, inside the device structure [4,16]. Finally, very recently, plasmonic circuits based on well-defined nano and micro-metallic patterns have been proposed as a new benchmark to build optical computers [17,18].

The optical properties of such organized plasmonic materials are complex and difficult to describe theoretically since both the intrinsic properties of the nanostructures and the long-range organization must be taken into account [19]. Generally, two main approaches are used to calculate the optical properties of plasmonic systems. The first approach consists in solving the Maxwell equations for a few nanostructures by defining their geometry explicitly. This approach relies on exact numerical simulations that require large computer resources, among which the most commonly used are discrete dipole approximation (DDA) [20,21], finite difference time domain (FDTD) [22] and boundary element method (BEM) [23,24]. These methods are very efficient for quite precisely computing both the optical spectra and the electric field maps of any nanostructure placed in a given medium. However, the computing time rapidly increases with the size of the system, thus limiting these methods to the study of very small nanoparticle assemblies [25]. The second approach is analytical and relies on the effective medium theories [26], the most famous being the Maxwell–Garnett (MG) theory [27]. Unlike the previous approach, the effective-medium phenomenological theories make it possible to tackle nanocomposites consisting of a large number of nanostructures. However, they proceed by averaging sizes, shapes, and orientation distributions and thus usually smooth out the intrinsic features of the nanoparticles. 

**Figure 1:**
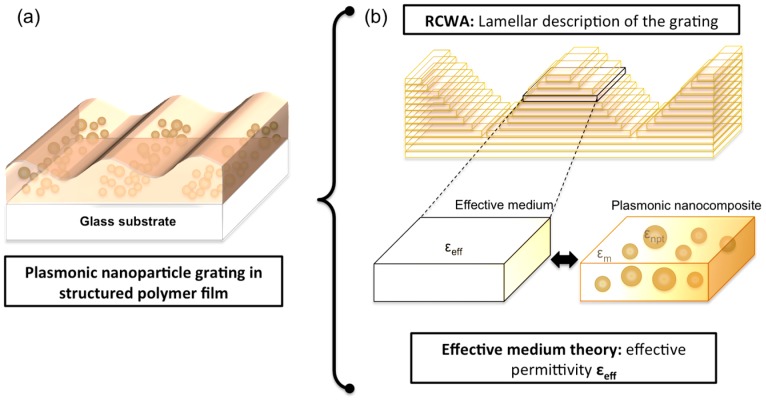
(**a**) Gold nanoparticle gratings in polymer thin films and (**b**) modeling of the system by combining the RCWA and the effective medium theory.

In the present work, we propose a new method for describing the optical properties of plasmonic systems with a multi-scale spatial organization consisting of ensembles of plasmonic nanostructures organized at the micrometer range. As a benchmark, we use gold nanoparticle gratings (GNGs) fabricated by laser interference patterning. In these systems, assemblies of nanoparticles are distributed in a polymer matrix of 1D gratings with a period of 2 µm (see Figure 1a).

We have prepared several gratings with a variable gold/polymer ratio and studied the effect of the gold volume fraction on the optical properties. Due to their specific structure, exact simulation of such gratings is impossible with the usual approaches such as DDA, FDTD, or BEM. Indeed, it would be necessary to consider a very large number of nanoparticles (several hundreds) to correctly describe their periodical spatial distribution at the micrometer scale. The computing time, along with memory allocation requirements, would become prohibitive. Consequently, there is a real need to develop new methods in order to address such systems. For that, we adopt an intermediate approach combining the semi-analytical MG theory and the so-called numerical method Rigorous Coupled Wave Analysis (RCWA) [28,29]. The periodic nature of the system is dealt with using the RCWA, which makes use of a stratified description of the composites, each stratum being described by its dielectric permittivity. The plasmonic properties of the nanocomposites are then introduced by replacing the dielectric permittivity of each stratum by an effective permittivity computed from the extended MG model. The modeling procedure is sketched in Figure 1b. This method allows us to compute the optical properties of the film and in particular the diffraction, the plasmonic nanostructures being implicitly described. 

## 2. Materials and Methods

### 2.1. Fabrication of Gold Nanoparticle Gratings

We briefly present the fabrication approach used to synthesize the GNGs. The detailed fabrication procedure is given in [30] and is summarized in Section A, Figure S1 of the Supplementary Materials. We have prepared three gratings with gold/polymer ratios of 3 wt %, 5 wt %, and 10 wt %. A polyvinyl alcohol (PVA) solution containing the appropriate amount of gold precursors is spin-coated on glass substrates. The films thus obtained are homogeneously doped with gold ions. Then, a Mach Zehnder interferometer is used to irradiate the sample with an interference pattern, thus performing a spatially controlled photo-reduction of the gold salt. Finally, nanoparticle growth is triggered by annealing the sample. It turns out that the nanoparticles are mainly formed in the irradiated areas (bright fringes of the interference pattern) and induce the deformation of the polymer film, leading to the appearance of sinusoidal surface relief gratings. A mechanism explaining the surface deformation has been proposed in [31]. As a comparison, another set of samples is prepared using the same procedure except for the irradiation part, which is performed with a homogeneous Gaussian beam instead of the interference pattern. Consequently, at the end of the procedure, the samples thus irradiated are loaded with gold nanoparticles homogeneously distributed in the films. This second set of samples will be denoted as homogeneous films (HFs). 

**Figure 2:**
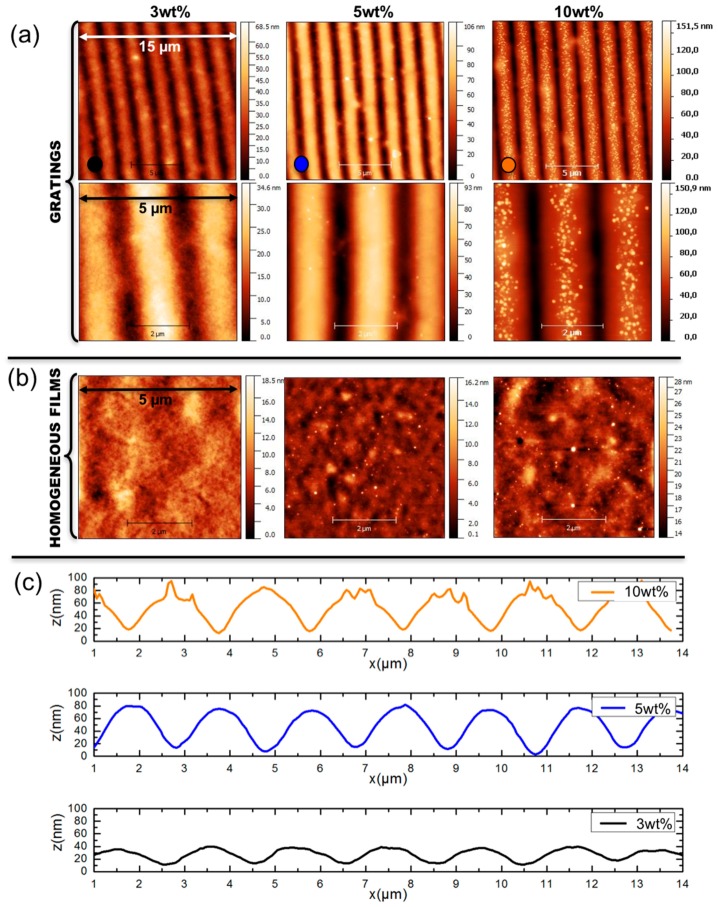
AFM images of the GNGs (**a**) and the HFs (**b**) with gold/polymer ratio of 3 wt %, 5 wt %, and 10 wt %. The surface profiles of the gratings are shown in (**c**).

The obtained films have an average thickness of 800 nm. Since scanning Electron Microscopy imaging becomes rather involved when dealing with dielectric media deposited on glass substrates, we have used Atomic Force Microscopy (AFM) to characterize the structural properties of our samples. AFM images of the various GNGs and the HFs with gold/polymer ratio of 3 wt %, 5 wt %, and 10 wt %, along with the surface profile of the gratings are given in Figure 2. As can be seen, the height of the surface modulation of the gratings increases with the gold concentration, varying from 25 nm to 80 nm, whereas the HFs remain flat at all concentrations. For low gold ratios, the nanoparticles are embedded in the films, but become clearly visible at the film surface as the gold concentration increases. In the case of the grating with 10 wt % gold, the nanoparticles start emerging at the top of the grating crests, which results in an uneven surface profile.

Absorption spectra have been measured on all samples by using standard spectroscopic techniques. A dedicated setup [31] has been used to measure the diffraction efficiency of the gratings, details are given in the Supplementary Materials (Section A, Figure S2).

### 2.2. Analytical and Numerical Models

In this section, we first introduce our approach for computing the dielectric permittivity of the gold nanoparticles. We then describe the extensions of the MG model that we have used to calculate the effective permittivity of the nanocomposites. Finally, we explain how the HFs and the GNGs are parameterized within the RCWA approach.

We have used two different MG models to compute the effective properties of the composites. The material is described as an ensemble of inclusions (the gold nanoparticles) randomly distributed, with a given volume filling fraction f, in a given host matrix (the polymer). The inclusions and the matrix are described with the help of their respective dielectric permittivities $\epsilon_{incl}$ and $\epsilon_{m}$. In the visible range, the permittivity of the polymer can be assumed to be constant and equal to $\epsilon_{m}=n_{PVA}^{2}=2.25$. On the other hand, the permittivity of the inclusions strongly depends on the wavelength. The most common way to model this behavior is to use the Drude model [30] or the tabulated data measured for bulk gold [32]. The latter option is usually preferred as it offers the possibility to account for the inter-band electronic transitions that take place in gold for wavelengths below 450 nm [33]. Even if this approach has proven to be fairly reliable, it has been pointed out that it is not fully appropriate for describing the behavior of gold at the nano-scale, especially in very small nanostructures. More precisely, when the size of the nanoparticles is reduced below the mean free path of the electrons, typically 40 nm in gold [34], the latter are strongly scattered by the nanoparticle surface, thus influencing their behavior. This effect, known as confinement [35], can be taken into account by introducing a damping term in the Drude model, depending on the nanoparticles radius R and a phenomenological parameter A. Based on this observation, it has been shown that very good agreement can be reached by modifying the tabulated data for bulk gold to take into account the effect of confinement. Consequently, the expression obtained in [36] for the permittivity of nanometric metallic inclusions is given by

(1) $$\epsilon_{incl}=\epsilon_{bulk}-\frac{\omega_{p}^{2}}{\omega^{2}+i\omega \left(\gamma +A\frac{v_{F}}{R}\right)}+\frac{\omega_{p}^{2}}{\omega^{2}+i\omega \gamma },$$

where $\epsilon_{bulk}$ is the tabulated permittivity of bulk gold [32], $\omega_{p}$ the plasmon pulsation of gold, γ a phenomenological damping coefficient [33], $v_{F}$ the Fermi velocity [33] and A a coefficient that is introduced for adjusting the effect of confinement. Various values of A can be found in the literature [37,38] and here we have chosen A=1 [39,40].

Equation (1) is then used to describe the effective permittivity of a nanocomposite material according to the standard MG model, which will be referred to as MGc (‘c’ stands for ‘confinement’). For a volume fraction f of inclusions, the effective permittivity can be calculated with the help of the following expression

(2) $$\epsilon_{eff}=\epsilon_{m}+3f\epsilon_{m}\frac{\epsilon_{incl}-\epsilon_{m}}{\epsilon_{incl}+2\epsilon_{m}-f\left(\epsilon_{incl}-\epsilon_{m}\right)}.$$

In fact, this model is only valid under the following conditions: (i) the concentration of the nanocomposites is small enough, i.e., the volume fraction is typically less than 10% and (ii) the nanoparticle radius falls in the range of 1–25 nm. The second condition reflects the fact that the MG model does not explicitly account for the nanoparticle size. However, experimentally it turns out that the nanoparticle size, and more precisely the size distribution of the nanoparticles assembly, may greatly affect the plasmonic response. To tackle this issue, extensions of the MG model, based on the Mie theory have been proposed in order to take into account the effect of particle size. Among them, the Modified Mie–Maxwell–Garnett model (MMMG), also introduces the effect of the nanoparticles size distribution [36,40]. Consequently, we compared the MGc model with this theory, together with Equation (1) accounting for the effect of confinement, and computed the effective permittivity of the gold nanocomposites. This model is referred to MMMGc and is given by

(3) $$\frac{\epsilon_{eff}-\epsilon_{m}}{\epsilon_{eff}+2\epsilon_{m}}=\frac{f}{\int_{R_{min}}^{R_{max}}R^{3}g\left(R\right)dR}\int_{R_{min}}^{R_{max}}\alpha_{Mie}\left(R\right)g\left(R\right)dR,$$

where, g(R) is the nanoparticle size distribution, with $R_{min}$ and $R_{max}$ its lower and upper bounds. Next,

(4) $$\alpha_{Mie}\left(R\right)=\frac{3i\lambda^{3}}{16\pi^{2}{\epsilon_{m}}^{\frac{3}{2}}}a_{1}\left(R\right),$$

is the nanoparticle polarizability with $a_{1}\left(R\right)$ being the first Mie (electric dipole) coefficient [41]. Note that here we ignore the (usually) weak magnetic contribution.

**Figure 3:**
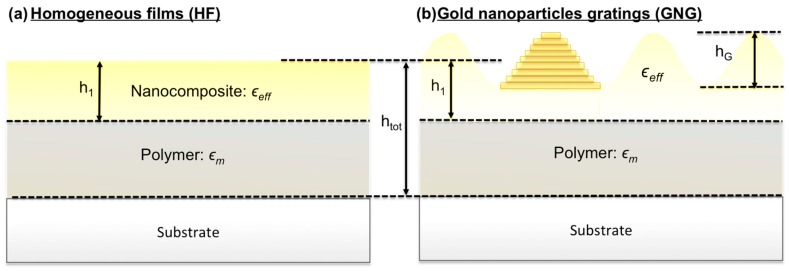
Parameterization of (**a**) the HFs and (**b**) the GNGs in the frame of the RCWA method.

We then use the RCWA approach to model the gratings as a periodic distribution of nanostructures. The parameterization of the system is sketched in Figure 3. We introduce the parameter $h_{1}$ as the thickness of the nanocomposite layer; it can be smaller than the total thickness of the film $h_{tot}$. In other words, the parameters $h_{1}$ determines the localization of the nanoparticles in the film thickness. Indeed, Scanning Electron Microscopy (SEM) measurements performed on the cross section of samples deposited on silicon wafers have shown that in some cases, the gold nanoparticles are located only in a thin layer on the polymer film surface (see Figure S8, Section B of the Supplementary Material). 

The HFs are defined as multilayer structures composed of the glass substrate, a layer of pure polymer (of thickness $h_{tot}-h_{1}$) and a layer of nanocomposite (of thickness $h_{1}$), whose effective permittivity $\epsilon_{eff}$ is computed using either the MGc or the MMMGc model as previously described. The volume fraction f of inclusions in the nanocomposite layer is normalized to ensure the conservation of the total mass of gold.

In the case of GNGs, we use the same parameterization except that the nanocomposite layer is now regarded as a succession of very thin layers in order to reproduce the surface profile of the grating. The surface profiles are assumed to be perfectly sinusoidal with an amplitude $h_{G}$ (see Figure 3b), so that the total volume of the nanocomposite layer is the same as that of a flat film defined by the same value of $h_{1}$. Consequently, the normalization of the volume fraction is the same as for HFs. 

## 3. Results and Discussion

We now compare the experimental spectra with the results of our calculations. In order to validate our theoretical method, we used as benchmarks the HFs and GNGs previously described in Section 2.1. It should be emphasized that the GNGs exhibit a novel plasmon-enhanced diffraction effect that was previously reported in [30]. This effect was defined as a resonant increase of the diffraction efficiency in the plasmon resonance domain, which can be quantified by plotting diffraction efficiency curves. The absorption and diffraction efficiency spectra have been simulated using the RCWA method as detailed in Section 2.2, the dielectric permittivity of the composites being computed using either the MGc (Equation (2)) or the MMMGc (Equation (3)) model.

We first discuss the results for the HFs. For our simulations we use the experimental values for f (calculated from the Au/PVA ratio). Indeed, the experimental absorption spectra do not present the characteristic peak of the gold precursor (at 320 nm), indicating that the latter has been entirely converted to metallic gold. The experimental spatial distribution of the nanoparticles in the film thickness could not be confirmed by structural characterizations. However, our simulations for HFs show that the parameter $h_{1}$ has a non-negligible effect on the absorption spectra, especially in the long-wavelength domain. More precisely, as $h_{1}$ increases, which is equivalent to having the nanoparticles distributed in a thicker layer, the agreement between the measured and simulated spectra improves, and the best fit is obtained for $h_{1}\;=\;h_{tot}$, i.e., when the nanoparticles assembly is spread over the total layer thickness (see section B, Figure S6 of the Supplementary Materials). Based on this result, we may conclude that the nanoparticles are evenly dispersed in the film. More generally, the effect of varying $h_{1}$ indicates that the nanoparticle organization within the hosting matrix plays a crucial role in the optical response for it affects the various scattering processes and interactions among the nanoparticles. A more quantitative analysis and comparison between theory and experiments would require, on one hand, a precise measurement of the nanoparticles depth, and taking account of the multi-scattering processes on the other.

**Figure 4:**
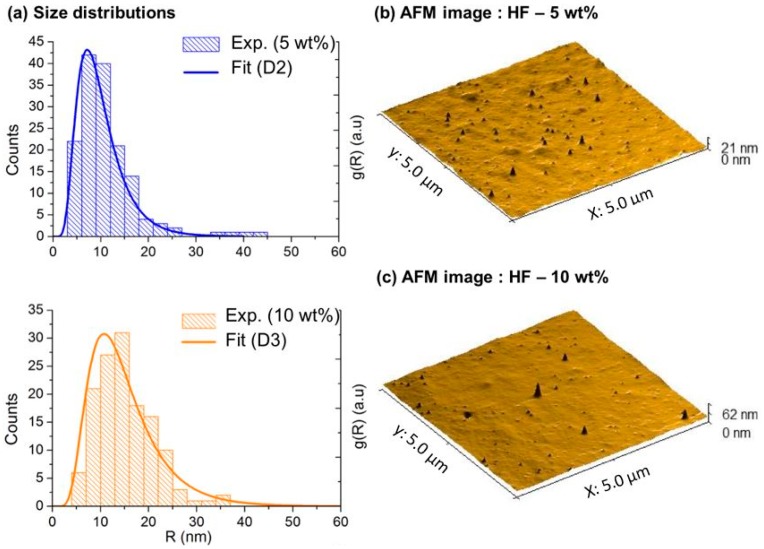
(**a**) Histogram and fitted size distribution of the HFs at 5 wt % and 10 wt %; (**b**) 3D view of typical AFM images of these samples.

**Figure 5:**
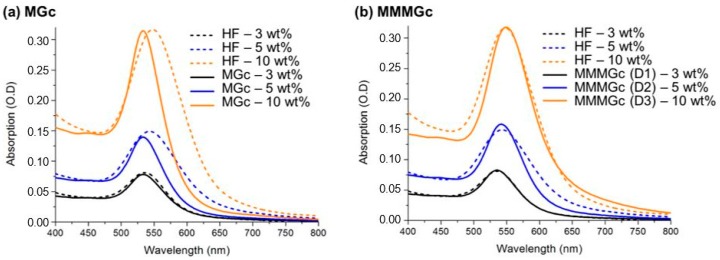
Comparison between the absorption spectra from experiments (dotted line) and simulations (continuous line) using the MGc model (**a**) and the MMMGc model (**b**).

Consequently, we have used $h_{1}=h_{tot}=800$ nm, assuming that the nanoparticles are evenly distributed in the total thickness of the films. For the MMMGc model, it is necessary to take into account the size distribution of the nanoparticles (see Equation (3)). In the case of the samples with 5 wt % and 10 wt % gold, experimental size distributions have been built by measuring a large number of nanoparticles that are visible on the AFM images of the film’s surface (tip effects were estimated and taken into account when evaluating the nanoparticles size). They have then been fitted with a lognormal distribution leading to the size distribution function g(R) used in the MMMGc model. Typical AFM images and the corresponding distributions for these samples are shown in Figure 4. It was not possible to apply the same procedure to the HF at 3 wt % because no nanoparticles were visible on the film’s surface in the AFM images. In the absence of experimental data, the size distribution was numerically optimized by fitting the absorption spectrum to obtain the best agreement between the simulated and the experimental data. The size distribution used for the sample at 3 wt % is given in the Supplementary Materials (Section B, Figure S5). For the MGc model, the effect of confinement, which depends explicitly on the nanoparticle size, was introduced by considering the mean value of the particle size distribution. Measured and simulated absorption spectra of the nanocomposites with Au/PVA ratio of 3 wt %, 5 wt %, and 10 wt % are given in Figure 5. 

The results show that the predictions of the MGc model are slightly blue-shifted compared to the experimental data. However, the MMMGc model with size distribution leads to a good agreement with the experimental data. Finally, the good agreement observed for plasmon resonance amplitude between simulations and experiments confirms the fact that the total amount of gold precursor introduced in the system has been reduced to metallic state during the fabrication procedure.

In summary, for simple systems such as HFs, our approach renders fairly good results, but it also shows that it is necessary to take account of the nanoparticles’ size distribution in the calculation of the effective permittivity.

**Figure 6:**
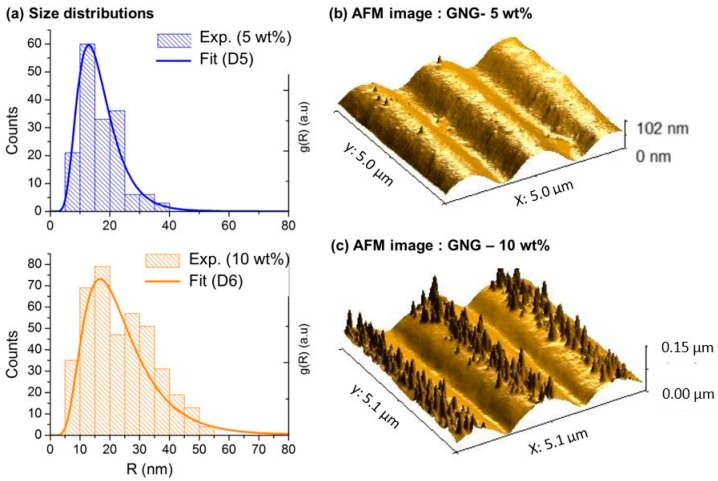
(**a**) Histograms and fitted size distributions of the GNGs at 5 wt % and 10 wt %; (**b**) 3D view of typical AFM images of these samples.

Next, we analyze the results for the GNGs. As before, the total thickness of the film, $h_{tot}$ = 800 nm, is the same for all samples and we have used values of the volume fraction f calculated from the experimental gold concentrations. The gratings heights, inferred from the surface profiles measured by AFM (see Figure 2b), are $h_{G}$ = 25 nm, 65 nm and 80 nm, respectively for the samples with Au/PVA ratio of 3 wt %, 5 wt %, and 10 wt %. In contrast with HFs, here we have found that the localization of nanoparticles in GNGs, described with the help of the numerical parameter $h_{1}$, plays a crucial role in the simulated spectra, in particular in what regards the diffraction properties. Indeed, as the periodic surface modulation is only superficial ($h_{G}\approx h_{tot}/10$), the concentration of the nanoparticles in this region strongly affects the diffraction efficiency of the system. Consequently, for each gold concentration, the parameter $h_{1}$ has been optimized in order to obtain the best agreement between the simulated and experimental spectra. The optimized values, summarized in the Supplementary Materials (Section B, Figure S9), are around 100 nm, which indicates that, contrary to the case of HFs, the nanoparticles are strongly localized near the film surface. From the fabrication point of view, it is likely that the localization of gold occurs during the irradiation process. As the gold ions experience the light intensity gradient of the interference pattern, they migrate toward the bright fringes and are photo-reduced near the surface of the films [42]. As for HFs, the nanoparticle size distribution used in the MMMGc model has been determined from AFM images for the nanocomposites at 5 wt % and 10 wt % (see Figure 6), and numerically optimized for the nanocomposite at 3 wt % (the final distribution is given in Figure S5 of the Supplementary Materials). The values of $h_{1}$ and all other simulation parameters for the GNGs are summarized in the Supplementary Materials (Section B, Figure S9).

**Figure 7:**
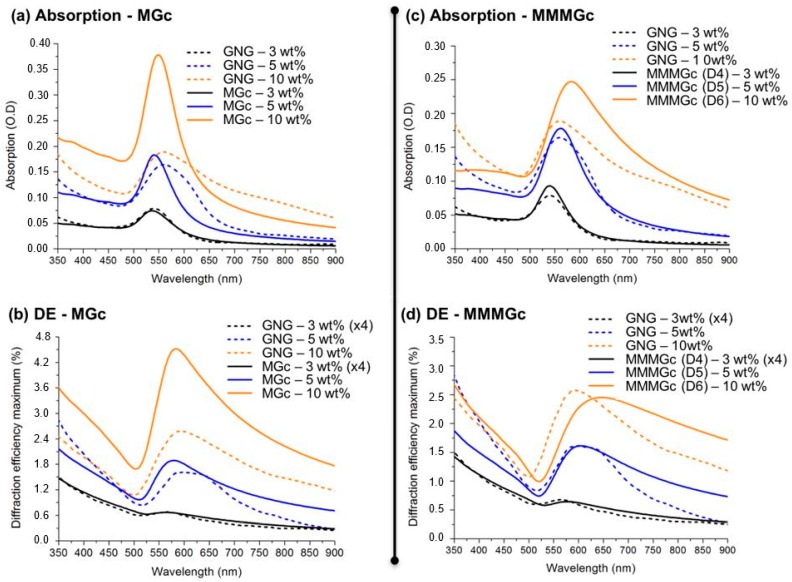
Comparison between the absorption spectra and the diffraction efficiency (DE) spectra measured experimentally and simulated using the MGc model (**a**,**b**) and the MMMGc model (**c**,**d**).

The experimental and simulated absorption and diffraction efficiency spectra are given in Figure 7. The first important result is the fact that our approach correctly accounts for the optical behavior of the GNGs, and in particular enables to reproduce the plasmon-enhanced diffraction effect. Upon closely analyzing the details, we find that the simulations show that, except for low gold concentrations (3 wt %), the MGc model does not provide a quantitative agreement with the experimental data. Indeed, the simulated absorption curves for the samples with 5 wt % and 10 wt % gold exhibit plasmon resonance profiles that are both narrower and blue-shifted, as compared to the measured ones. The same conclusions can be drawn for the diffraction efficiency spectra, which, even if they show the right tendency, do not allow for quantitative predictions. On the other hand, the results for the MMMGc model show that taking into account the particle size distribution qualitatively and quantitatively improves the results, in particular for the sample with 5 wt % gold. However, for 10 wt % gold, even if there is a better qualitative agreement between theory and experiment than for the MGc model, the discrepancies are still large in both absorption and diffraction efficiency spectra. In particular, with this approach, we cannot precisely predict the position of the plasmonic peaks at this concentration.

**Figure 8:**
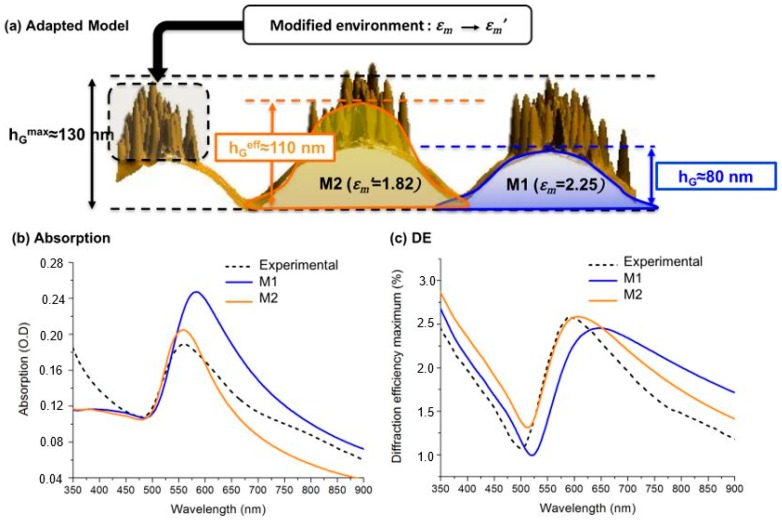
Specific consideration for the GNG at 10 wt %. (**a**) Parameters for the new modeling approach (M2) and the previous one (M1). (**b**) Absorption and (**c**) Diffraction efficiency (DE) spectra of the GNG: comparison between the experimental data and the two models.

The latter observation is, however, not quite surprising. Indeed, if we analyze the AFM images of the GNG at 10 wt % gold (see Figure 6c), we notice a very high density of nanoparticles at the grating crests emerging from the surface. This means that the nanoparticles are not fully embedded in the polymer matrix anymore, so that our simulations based on the effective medium approach are not really suitable for describing the system. For a better description of the emerging nanoparticles, we proposed a new parametrization of the system (M2) sketched in Figure 8a. First, we increase the grating height in order to include, at least partially, the emerging nanoparticles inside the simulated nanocomposite layer. The effective grating height $h_{G}^{eff}$ has been varied between 80 nm ($h_{G}$) and 130 nm, which corresponds to the maximal height that is reached only locally by some nanoparticles. We have found that this parameter strongly affects the simulated spectra, in particular the amplitude of the diffraction efficiency and the best agreement with the experimental spectra is obtained for $h_{G}^{eff}=110$ nm. Second, since the nanoparticles are partly covered by polymer on one side and by air on the other, they experience a different environment, which may be characterized by a smaller value of the matrix permittivity. Numerical optimization leads to $\epsilon_{m}$ =1.82 (i.e., n = 1.35 instead of 1.5). The results obtained with this new model (M2) are compared with the previous ones (model M1, $h_{G}$ = 80 nm, $\epsilon_{m}$ = 2.25) in Figure 8b,c.

It is clear that the new set of parameters, which corresponds to a more plausible physical description of the GNG, improves the agreement between experiments and theory. We see in particular a better prediction regarding the resonances position and amplitude in both absorption and DE spectra. These two effects are mainly due to the change of environment (modified $\epsilon_{m}$), since the plasmon resonance of nanoparticles becomes more blue-shifted and weaker in magnitude when the refractive index of their surrounding medium decreases. It can be seen that, even with this improved model, we cannot reproduce the shoulder appearing on the experimental spectra around 750–800 nm. This feature, which is not observed for the other samples, may be attributed to plasmonic coupling between strongly packed nanoparticles [43]. Indeed, at the grating crests, the nanoparticle concentration can be locally very high. As a consequence, aggregates may appear and involve complex multi-polar coupling among the nanoparticles. Unfortunately, such effects cannot be described properly by the effective medium approach used here, which only includes the dipolar coupling. 

## 4. Conclusions

In conclusion, we have fabricated nanocomposites in the form of homogeneous films (HFs) and gold nanoparticle gratings (GNGs) by using an in situ synthesis approach under laser irradiation. We have measured and simulated both the absorption and the diffraction efficiency spectra of these systems with a variable gold/polymer ratio. We have shown that the combination of RCWA and (extended) effective medium theories is a promising means for computing the optical properties of complex plasmonic systems. In particular, our simulations reproduce the previously observed effect of plasmon-enhanced diffraction in GNGs. For polydisperse nanoparticles, it has been shown that the size distribution must be taken into account in order to reach a quantitative agreement with experiment. Finally, we have speculated how the present model may be adapted so as to describe the specific configuration of GNGs at 10 wt %. This demonstrates the versatility of this approach and shows how it can be used for a better description of the physical behavior of structured plasmonic nanocomposites. Our approach captures most of the physics underlying the optical properties of nanocomposite films and also applies at high nanoparticles concentrations, even if it does not reproduce high frequency features appearing in the case of highly packed nanoparticles. We believe that this method, that is rather simple to implement and does not require substantial computational power, provides us with an efficient tool to study multi-scale plasmonic systems that cannot be dealt with using standard numerical approaches. For instance, it could be used to study the original optical properties that show up in metamaterials based on block-copolymer self-assembly [2,15,44]. In addition, for numerous nanocomposite systems, direct visualization of the inner structure of the material with the usual characterization techniques, such as SEM or TEM (Transmission Electron Microscopy), can be quite challenging. In such a situation, our approach could be used to investigate the nanoparticle size distribution and localization in the material upon fitting the experimental absorption spectra. 

## Supplementary Materials

The following are available online at http://www.mdpi.com/1996-1944/11/3/351/s1. Figure S1: Main steps of the fabrication method; Figure S2: Absorption of the HFs and the GNGs with Au/PVA ratio of 3 wt %, 5 wt %, and 10 wt %; Figure S3: Spectro-goniometer, setup used for the measurement of diffraction efficiency spectra; Figure S4: DE maps of the GNGs with Au/PVA ratio of (a) 3 wt %, (b) 5 wt %, and (c) 10 wt % and the corresponding DE spectra (d); Figure S5: Distributions obtained from numerical optimization for the HF and the GNG at 3 wt %; Figure S6: Effect of nanoparticles localization on the absorption spectra of a HF at 10 wt %; Figure S7: Parameters used for the simulation of HFs with Au/PVA ratio of 3 wt %, 5 wt %, and 10 wt %; Figure S8: SEM images of GNG cross section showing the localization of the nanoparticle at the film surface; Figure S9: Parameters used for the simulation of GNGs with Au/PVA ratio of 3 wt %, 5 wt %, and 10 wt %.
