# Supplementary material for: Optical Properties of Complex Plasmonic Materials Studied with Extended Effective Medium Theories Combined with Rigorous Coupled Wave Analysis
Source: Materials (Basel). 2018 Feb 27;11(3):351. doi: 10.3390/ma11030351 (PMC5872930; doi:10.3390/ma11030351)
Supplement: Supplementary file 1 [file materials-11-00351-s001.pdf]

Supporting Material

Optical properties of complex plasmonic materials studied with extended effective medium theories combined with the Rigorous Coupled Wave Ananlysis

Elie Nadal[1,2], Noémi Barros[1,2], Hervé Glénat[2], Hamid Kachakachi[1,2]

[1] University of Perpignan Via Domitia (UPVD), 52 Avenue Paul Alduy, 66100 Perpignan, France
[2] PROMES, CNRS (UPR8521), Rambla de la thermodynamique, 66100, Perpignan, France.

Corresponding author: elie.nadal@univ-perp.fr

A. Experimental details

1. Sample fabrication

The GNGs are fabricated according to the steps described in Fig.S1:

1. We first prepare a polymer solution with a concentration of 100 mg/ml by heating Polyvinyl alcohol (PVA, Mw 13000-23000, 89% hydrolyzed, Aldrich) at reflux in deionized water. Appropriate amounts of gold chloride ($HAuCl_4$, $3H_20$, 99% Pure, Aldrich) are dissolve in the polymer solution under vigorous stirring so as to obtain metal/polymer blends with ratio of 3%, 5% and 10% in weight (w%).
2. The films are then deposited by spin coating (1500 rpm for 60 s) on cleaned glass substrates. The final height, measured with a profilometer is around 800 nm. We use a Mach-Zehnder interferometer to irradiate the film with an interference pattern (λ=473 nm, grating step = 2 µm, light flux = 100 mW/cm^2) thus performing a localized photo-reduction of the gold salt.
3. The films are finally heated at 130°C during 3 minutes on hot plate, which induces the nanoparticles growth and the surface relief grating formation.

The preparation of the HFs follows the same procedure except that the irradiation is performed by using a homogeneous Gaussian beam with the exact same intensity (100 mW/cm^2).

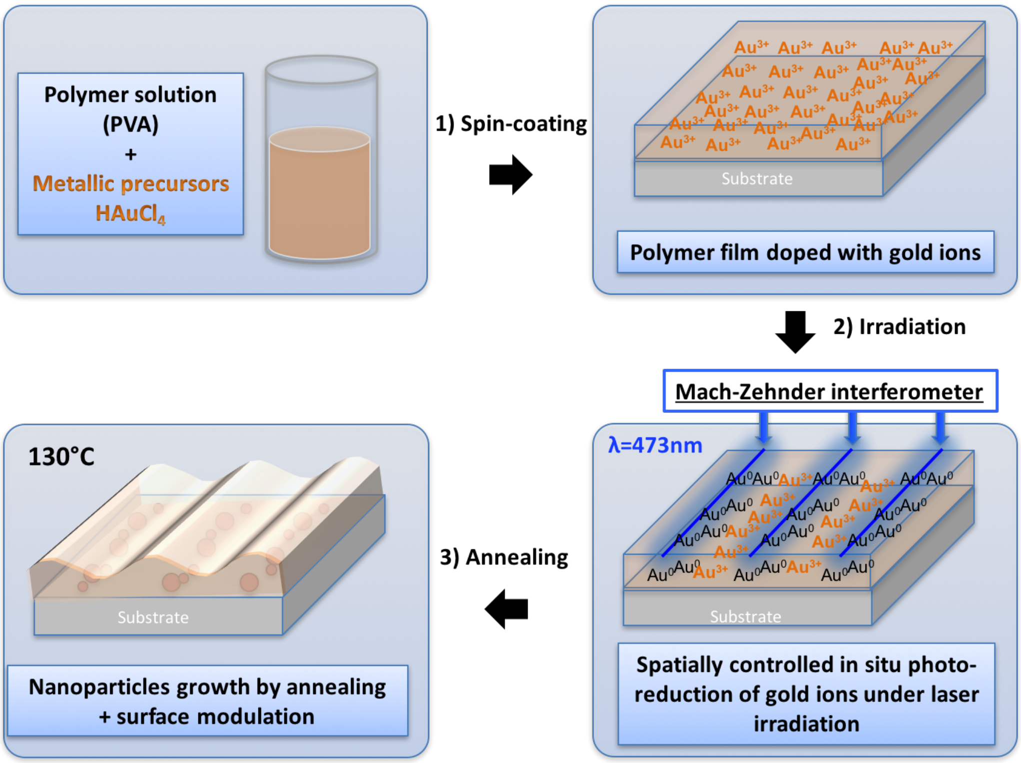

Figure S1: Main steps of the fabrication method.

2. Optical measurements

The optical properties of the films are then characterized. We first measure absorption spectra by using standard spectroscopic techniques. The results for the GNGs and the HFs are given in Fig.S2.

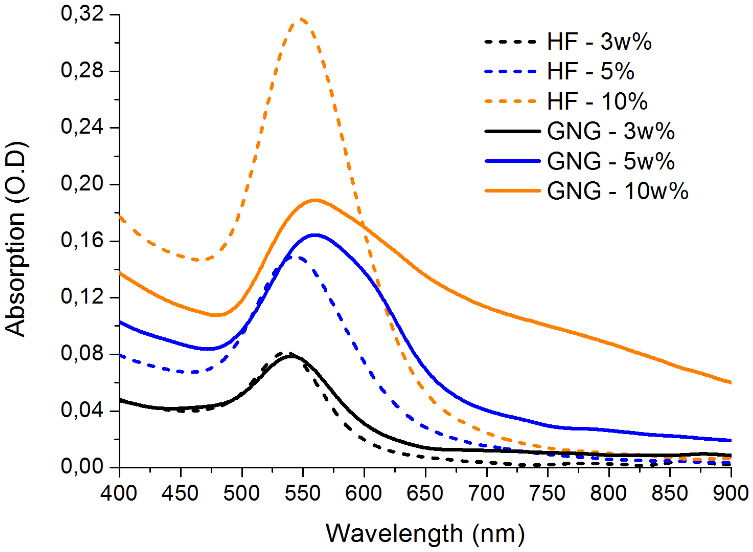

Figure S2: Absorption of the HFs and the GNGs with Au/PVA ratio of 3w%, 5w% and 10w%.

We then look at the diffractive properties of the gratings. We measure the spectral dependence of the diffraction by taking transmittance spectra at different angles with a spectro-goniometer, described in Fig. S3.

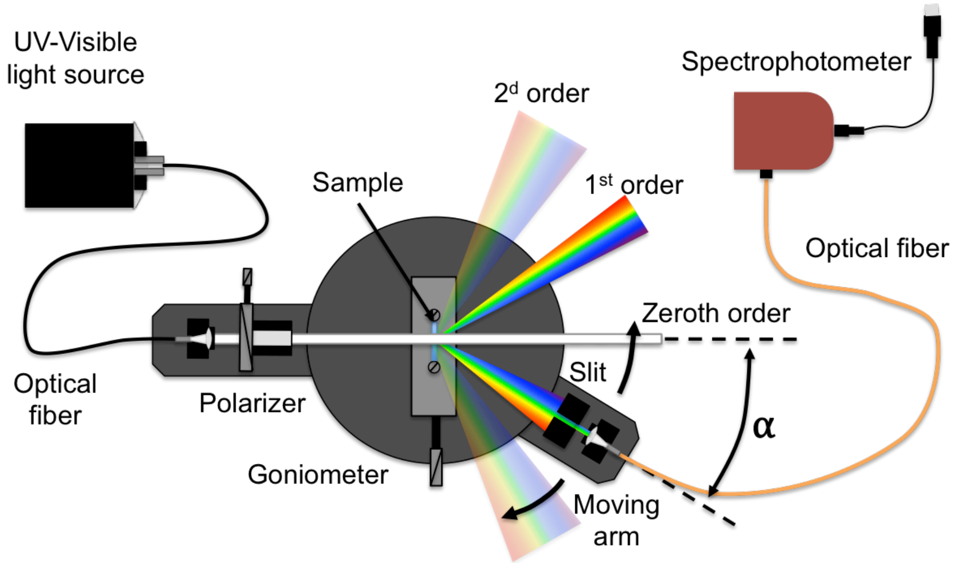

Figure S3: Spectro-goniometer, setup used for the measurement of diffraction efficiency spectra.

The spectra are normalized by the transmitted spectra of the zero order such that the measured diffraction efficiency is defined by

$$\eta(\alpha,\lambda) = \frac{I_i(\alpha,\lambda)}{I_0(0,\lambda)}$$

where $I_i(\alpha,\lambda)$ is the transmitted intensity of the ith order, $I_0(0,\lambda)$ the transmitted intensity of the zeroth order, α the angle at which the measurement is performed and λ is the wavelength of light. By combining these spectra, it is possible to plot diffraction efficiency (DE) maps as a function of wavelength and diffraction angle, see Fig.S4 (a), (b) and (c). Finally, the DE spectra are plotted by considering the maximum of the diffraction efficiency as a function of wavelength, independently of the angle (it corresponds to the regions framed by the orange dotted line in the figure). The spectra, given in Fig.S4.(d), correspond to the first diffracted order only.

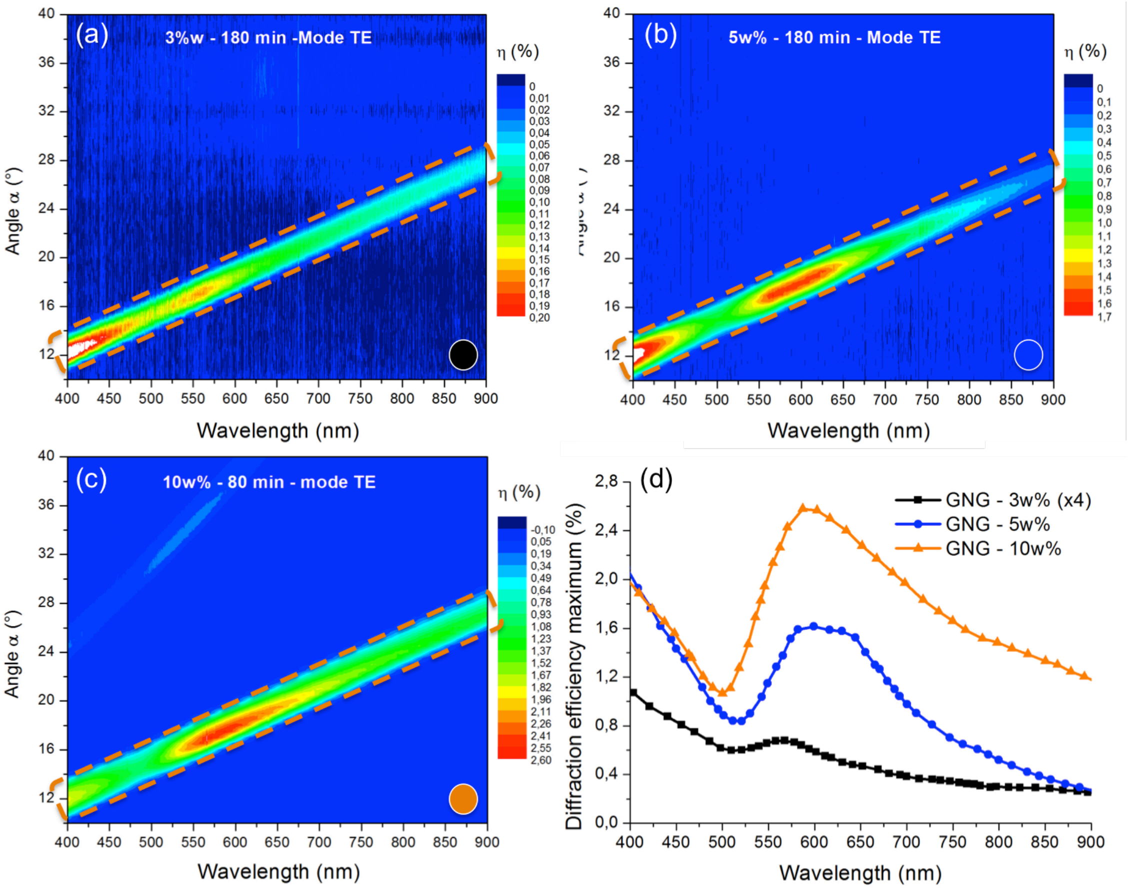

Figure S4: DE maps of the GNGs with Au/PVA ratio of (a) 3w%, (b) 5w% and (c) 10w% and the corresponding DE spectra (d).

B. Simulations

The aim of this section is to give additional details regarding the modeling of the HFs and the GNGs. In particular, all the simulation parameters used to simulate each of the samples are summarized.

Regarding the HF and the GNG at 3w%, the nanoparticle size distributions for the MMMGc model have been optimized numerically as it was impossible to estimate the distribution from AFM images. The distribution D1 and D4 respectively for the HF and the GNG are given in Fig.S5.

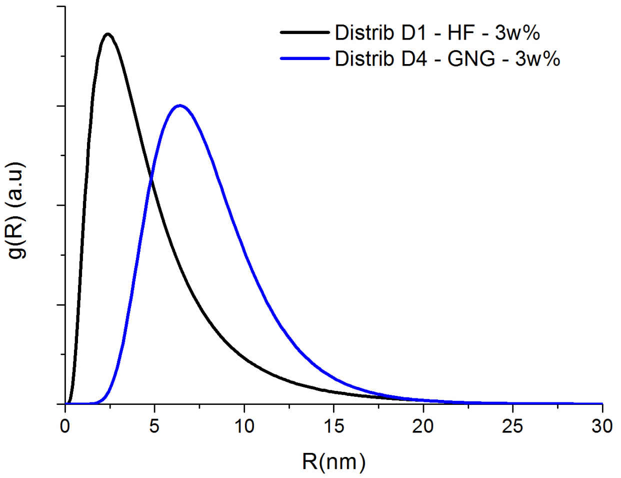

Figure S5: Distributions obtained from numerical optimization for the HF and the GNG at 3w%.

In order to study the effect of the nanoparticle localization in the film, driven by the parameter h_1, we performed a series of simulations for the HF at 10w% by varying h_1 from 800 nm (i.e. the total thickness of the film h_{tot}) down to 100 nm. As the nanoparticle are concentrated in a thinner layer close to the surface, the filling fraction f increases. The results are shown in Fig.S6.

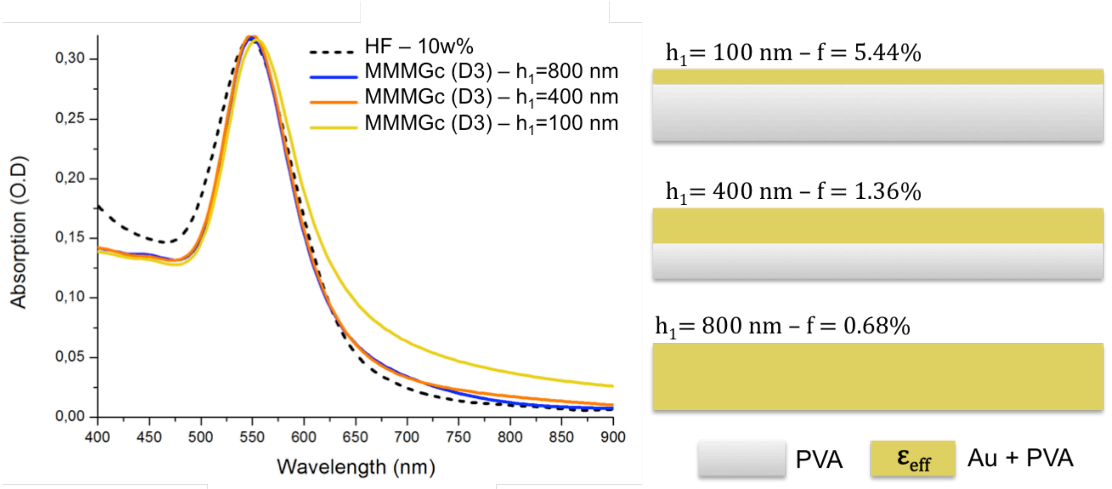

Figure S6: Effect of nanoparticles localization on the absorption spectra of a HF at 10w%.

The spectra show that, for HFs, the localization of the nanoparticles at the top of the film has only a limited effect on the absorption spectra. Consequently, we choose to perform our simulation by considering that the nanoparticles are homogeneously distributed in the polymer films ($h_1 = h_{tot}$).
All the simulation parameters used to compute the absorption spectra of the HF are summarized in the Fig.S7.

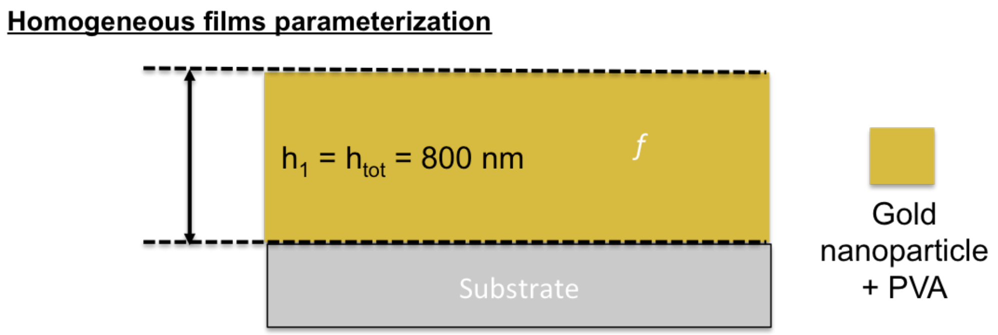

Au/PVA ratio	h_1 (nm)	*f* (V%)	ε_m
3w%	800	0.19	2.25
5w%	800	0.31	2.25
10w%	800	0.66	2.25

Figure S7: Parameters used for the simulation of HFs with Au/PVA ratio of 3w%, 5w% and 10 w%.

The case of GNGs is different. Indeed, the spatially controlled irradiation of the films seems to lead to the migration of the gold salt toward the surface during the photo-reduction process. Consequently, the nanoparticles accumulate in this region, thus forming the surface relief grating. This effect has been confirmed by measuring the cross-section of samples prepared on silicon wafers with SEM. Typical images are given in Fig.S8.

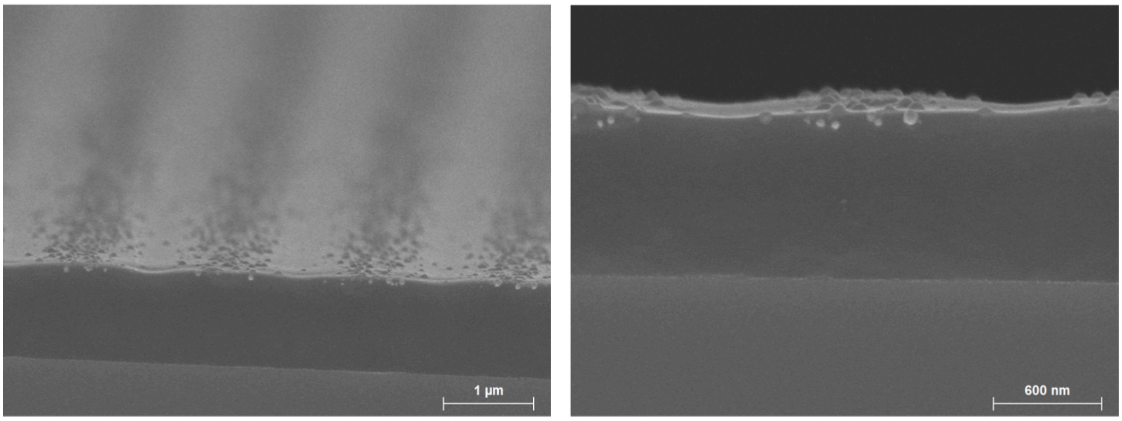

Figure S8: SEM images of GNG cross-section showing the localization of the nanoparticle at the film surface.

Consequently, in the case of GNGs, the parameter h_1 was numerically optimized to fit the experimental spectra. We found in particular that the density of nanoparticles located inside the sinusoidal grating will strongly influence the diffraction properties of the system, even at fixed value of h_R.
All the simulation parameters used to compute the absorption end the DE spectra of the GNGs are summarized in the Fig.S9.

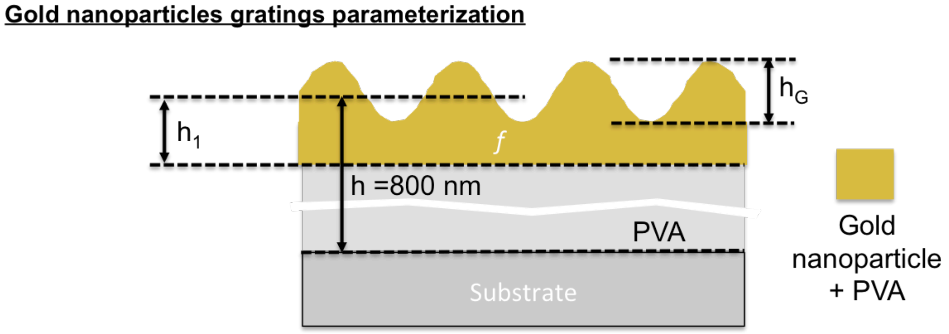

Au/PVA ratio	h_G (nm)	h_1 (nm)	*f* (V%)	ε_m
3w%	25	100	1.77	2.25
5w%	65	70	6.90	2.25
10w%	80	80	13.61	2.25
10w%	110	110	9.90	1.82

Figure S9: Parameters used for the simulation of GNGs with Au/PVA ratio of 3w%, 5w% and 10 w%.
